# Immunoregulation in Fungal Infections: A Review and Update on the Critical Role of Myeloid-Derived Suppressor Cells

**DOI:** 10.3390/jof11070496

**Published:** 2025-06-30

**Authors:** Valéria de Lima Kaminski, Ana Luiza Oliveira Menezes, Kauan Gonçalves de Lima, Stephani Leonelo de Almeida, Diego Vinícius Alves da Silva, Filipe Nogueira Franco, Nycolas Willian Preite, Flávio Vieira Loures

**Affiliations:** 1 School of Medicine, Universidade Anhembi Morumbi, Benedito Matarazzo Avenue, 6070, São José dos Campos 12230-002, SP, Brazil; valeria.lkaminski@gmail.com (V.d.L.K.); oliveiramenezesanaluiza@gmail.com (A.L.O.M.); kauangoncalveslima@gmail.com (K.G.d.L.); stephani.leonelo16@gmail.com (S.L.d.A.); dievinalves@gmail.com (D.V.A.d.S.); 2 Institute of Science and Technology, Universidade Federal de São Paulo (UNIFESP), Talim Street, 330, São José dos Campos 12231-280, SP, Brazil; filipenogueirafranco@gmail.com (F.N.F.); preite@unifesp.br (N.W.P.)

**Keywords:** immunosuppression, MDSC, fungi, *Candida* spp., *P. brasiliensis*, *Aspergillus* spp., *C. neoformans*, immunotherapy

## Abstract

Myeloid-derived suppressor cells (MDSCs) are a heterogeneous group of immune cells that play a central role in regulating host immune responses during fungal infections. Their recruitment is mediated by pathogen recognition receptors, particularly Dectin-1 and CARD9 signaling, which promote the production of reactive oxygen species (ROS) and IL-1β. Once activated, MDSCs suppress T-cell and natural killer cell functions through immunosuppressive cytokines like IL-10 and TGF-β, as well as enzymes such as arginase-1 and indoleamine 2,3-dioxygenase 1 (IDO-1). This review explores the role of MDSCs in fungal infections caused by *Candida* spp., *Paracoccidioides brasiliensis*, *Aspergillus* spp., and *Cryptococcus neoformans*, emphasizing their impact on immune modulation and disease progression. The emerging evidence suggests that fungal bioactive compounds, such as polysaccharides, can influence MDSC activity and restore immune balance. Notably, therapies targeting MDSCs have demonstrated promise in both fungal infections. In particular, infections with *P. brasiliensis* and *C. neoformans* show improved T-cell responses following MDSC-targeted interventions. Additionally, polysaccharides from *Grifola frondosa* and exposure to *Aspergillus sydowii* affect MDSC behavior, supporting the potential of modulating these cells therapeutically. Together, these findings highlight the relevance of MDSCs in fungal pathogenesis and underscore their potential as targets for immunotherapeutic strategies in infectious diseases.

## 1. Introduction

Fungal infections represent a growing public health risk, particularly in individuals with compromised immune systems, such as those infected with human immunodeficiency virus (HIV), patients undergoing immunosuppressive treatment, or post-transplant recipients [1,2,3]. Opportunistic fungi exploit immune system vulnerabilities, leading to severe infections that often exhibit high lethality rates. The mortality associated with opportunistic fungal infections can exceed 50%, with particularly high rates observed in post-transplant patients, as seen in cases of aspergillosis, mucormycosis, cryptococcosis, and candidiasis [4,5]. The severity of these infections is directly related to the fungi’s ability to evade the immune system through various mechanisms that interfere with the effectiveness of innate and adaptive immune responses [6].

Among the immune evasion mechanisms, fungi hinder phagocytosis due to the structure of their cell walls and induce the production of immunosuppressive cytokines, which suppress T-cell activation, such as interleukin-10 (IL-10), transforming growth factor-beta (TGF-β), and interleukin-35 (IL-35) [6]. Additionally, the polarization of the immune response toward a Th2 profile, which is less effective against fungi, at the expense of a protective Th1 response, further reduces the host’s ability to control the infection [6,7]. These microorganisms also interfere with dendritic cell (DC) function, impairing antigen presentation and T-cell activation, thereby facilitating fungal survival and proliferation, namely in immunocompromised hosts [8].

In this context of immunosuppression and altered immune responses, myeloid-derived suppressor cells (MDSCs) emerge as key regulators of immunity [9]. These heterogeneous cells are critical in modulating immune responses in several pathological conditions, including neoplasms, autoimmune diseases, and infections [9,10]. MDSCs are derived from myeloid precursors common to other cell types, such as monocytes and neutrophils, and remain in an immature differentiation state, being rare under physiological conditions. However, MDSCs are widely expanded in environments of chronic or acute inflammation [10,11].

MDSCs are classified into different subtypes based on cell surface markers. In mice, MDSCs are divided into two main subsets: monocytic MDSCs (M-MDSCs) and polymorphonuclear MDSCs (PMN-MDSCs), also called granulocytic MDSCs (G-MDSCs) [12]. M-MDSCs are defined as CD11b⁺ Ly6C^high^ Ly6G^−^, and PMN-MDSCs are characterized as CD11b⁺ Ly6Clow Ly6G⁺ in mice [13]. In humans, MDSCs are classified into three main subtypes: monocytic MDSCs (mMDSCs), characterized by HLA-DR−/low CD33⁺ CD11b⁺ CD14⁺; polymorphonuclear or granulocytic MDSCs (gMDSCs), identified by Lin^−^ HLA-DR^−/low^ CD33⁺ CD11b⁺ CD15⁺; and early-stage MDSCs (eMDSCs), which are Lin^−^ (including CD3, CD14, CD15, CD19, and CD56) and HLA-DR^−/low^ CD33⁺, reflecting their phenotypic and functional diversity in inflammatory environments [13]. The diversity of MDSC subtypes enables distinct immunosuppressive mechanisms, playing a crucial role in modulating inflammation and facilitating immune evasion across various pathological conditions. Beyond fungal infections [14], MDSCs have also been implicated in bacterial [15], viral [16], and parasitic diseases [17], highlighting their broad relevance in microbial pathogenesis.

MDSC-mediated immunosuppression occurs through mechanisms such as arginase-1 production in conjunction with high levels of iNOS, thereby limiting T-cell proliferation [9,13]. Simultaneously, the expansion of these cells is driven by inflammatory mediators, such as prostaglandin E2, pro-inflammatory cytokines (TNF-α, IL-6, and IL-1β), and the calgranulin B protein (S100A9), highlighting their relevance in pathological environments [18,19,20].

In fungal infections, the recruitment of MDSCs can occur in response to fungal recognition by innate immune cells through pathogen recognition receptors (PRRs), such as C-type lectin receptors (CRLs), Toll-like receptors (TLRs), and galectin family proteins [21,22,23]. The composition of the fungal cell wall changes according to the fungal species’ morphotype, stage of growth, and environmental conditions. It serves as the primary source of pathogen-associated molecular patterns (PAMPs), which are detected by PRRs on mammalian cells [23]. The most commonly recognized fungal PAMPs by host PRRs are key structural components of the fungal cell wall, which are conserved across medically relevant species. These include β-glucans—particularly β-(1,3)-glucans with varying degrees of β-(1,6) branching—chitin, a polymer of N-acetylglucosamine, and mannans, composed of mannose chains attached to fungal proteins via N- or O-linkages. In addition, β-(1,2)-linked oligomannosides serve as PAMPs and are specifically recognized by galectin-3, enabling phagocytes to distinguish between pathogenic and non-pathogenic yeasts [6,22].

Fungal pathogens are primarily recognized by host PRRs that detect conserved pathogen-associated molecular patterns (PAMPs) located in the fungal cell wall. Key PAMPs include β-glucans, mannans, and chitin, which vary in structure depending on the fungal species. C-type lectin receptors (CLRs), particularly Dectin-1, Dectin-2, and mincle, are central to sensing these components, with Dectin-1 being the main receptor for β-glucans, triggering downstream signaling via SYK–CARD9 and RAF pathways that coordinate cytokine production and inflammasome activation [24,25]. TLRs, such as TLR2, TLR4, and TLR9, also contribute to fungal recognition by detecting components like zymosan, phospholipomannan, and fungal DNA [21]. Additionally, NOD-like receptors (NLRs), including NLRP3, play a role in inflammasome assembly and IL-1β/IL-18 maturation during fungal infection, with knockout models highlighting their importance in host defense [26,27,28,29].

These mechanisms are crucial for initiating the inflammatory response and modulating the immune microenvironment. Once recruited, MDSCs exert a potent immunosuppressive effect by inhibiting T-cell and natural killer (NK) cell activation through the secretion of immunomodulatory cytokines such as IL-10 and TGF-β as well as through the expression of enzymes like arginase-1, which depletes L-arginine (an essential amino acid for T-cell activation and proliferation), and indoleamine 2,3 dioxygenase 1 (IDO-1), which reduces tryptophan availability, also leading to reduced T-cell proliferation [8,20,30,31].

It is also important to highlight that, in fungal infections, the role of MDSCs is highly dependent on the pathogen involved. In *Candida albicans* infection, MDSCs exert a protective effect by modulating the Th17 immune response, promoting host survival in murine experimental models [14]. In this context, Rieber et al. [14] demonstrated that neutrophilic MDSCs exerted a protective role in a murine model of systemic *C. albicans* infection, whereas no such effect was observed in pulmonary *Aspergillus fumigatus* infection. This finding highlights the differential role of MDSCs in fungal infections and supports their potential as a therapeutic approach in invasive *C. albicans* infections but not in those caused by *A. fumigatus*. The protective effect mediated by MDSCs was linked to the suppression of NK and T-cell activation as well as Th17 responses; notably, in vivo supplementation with IL-17A partially counteracted this effect. Based on these results, the authors propose that MDSCs may benefit the host by attenuating pathogenic hyperinflammatory NK and Th17 responses. Thus, strategies aimed at enhancing neutrophilic MDSCs could represent a novel anti-inflammatory approach in the treatment of fungal infections, particularly those involving *C. albicans* [14]. Thus, in diseases caused by *A. fumigatus*, *Paracoccidioides brasiliensis* [30], and *Cryptococcus neoformans* [32], MDSCs do not exhibit the same protective response, indicating that their immunomodulatory functions are contextual and depend on the specific characteristics of each pathogen.

This integrative review aimed to analyze the role of MDSCs in the immunoregulation of fungal infections by synthesizing the available evidence regarding their function in this context. In addition, we sought to identify potential therapeutic strategies based on the modulation of MDSCs and highlight the gaps in the literature that may guide future research on the interaction between MDSCs and fungal pathogens. Searches were performed in the PubMed, Virtual Health Library (VHL), and LILACS databases, using controlled descriptors and free terms combined with Boolean operators. Articles available in full text in English, Portuguese, and Spanish were included. For study selection, only experimental articles that investigated MDSCs in fungal infections or used fungi extracts to promote MDSC recruitment were included, emphasizing the interaction between these cells and fungi. Studies addressing the interaction between fungi and MDSCs in carcinogenesis and potential therapeutic strategies involving these cells were also considered.

Among the analyzed studies, seven addressed infections by *Candida* spp., highlighting the clinical relevance and high incidence of candidiasis. Four articles focused on paracoccidioidomycosis (PCM), caused by *P. brasiliensis*. At the same time, three studies focused on aspergillosis, emphasizing the pathogenic role of *Aspergillus* spp., and two articles focused on cryptococcosis, caused by the fungus *C. neoformans*. Furthermore, six articles explored strategies based on mushroom extracts and polysaccharides, aiming to modulate MDSCs in experimental models, as summarized in Table 1.

**Figure 1:**
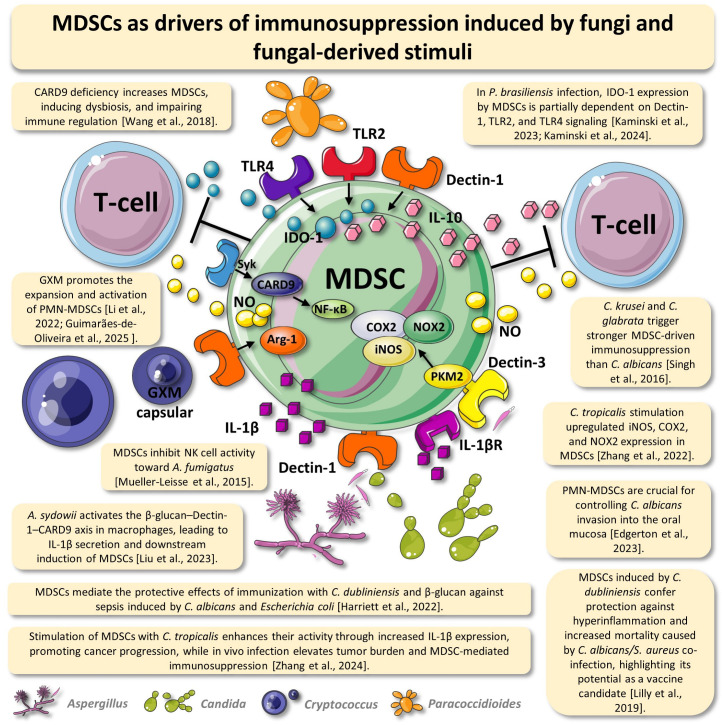
Myeloid-derived suppressor cells (MDSCs) as drivers of immunosuppression induced by fungi and fungal stimuli. This figure depicts a hypothetical MDSC at the center, illustrating its activation and effector mechanisms in response to fungal pathogens and their bioactive components. The MDSC is shown to express pattern recognition receptors, such as TLR2, TLR4, Dectin-1, and Dectin-3, which recognize fungi-associated molecular patterns and initiate intracellular signaling cascades involving CARD9, Syk, and NF-κB. These pathways lead to the production of immunosuppressive molecules, including nitric oxide (NO), interleukin-10 (IL-10), interleukin-1β (IL-1β), indoleamine 2,3-dioxygenase 1 (IDO-1), arginase-1 (Arg-1), inducible nitric oxide synthase (iNOS), cyclooxygenase-2 (COX2), NADPH oxidase 2 (NOX2), and pyruvate kinase M2 (PKM2). Fungal species surrounding the MDSC include *Paracoccidioides brasiliensis*, which induces IDO-1 expression via TLR2, TLR4, and Dectin-1 signaling; *Cryptococcus neoformans*, whose capsular glucuronoxylomannan (GXM) promotes the expansion of polymorphonuclear MDSCs (PMN-MDSCs); *Aspergillus sydowii*, which activates MDSCs through IL-1β signaling; and various *Candida* species, such as *C. krusei*, *C. glabrata*, *C. albicans*, *C. dubliniensis*, and *C. tropicalis*, which modulate MDSC function by enhancing the expression of immunosuppressive enzymes and cytokines. In particular, stimulation with *C. tropicalis* increases iNOS, COX2, NOX2, and IL-1β, contributing to MDSC-mediated suppression of T-cell and NK cell activity. Additional stimuli such as β-glucan and fungal polysaccharides also contribute to MDSC activation and the modulation of host immunity. Selected findings from the literature supporting these interactions are presented in callout boxes. The theoretical framework supporting the figure is described throughout the main text and is referenced in the figure’s annotated information boxes. Abbreviations: IDO-1—indoleamine 2,3-dioxygenase 1; IL—interleukin; NO—nitric oxide; Arg-1—arginase-1; iNOS—inducible nitric oxide synthase; COX2—cyclooxygenase-2; NOX2—NADPH oxidase 2; TLR—Toll-like receptor; PKM2—pyruvate kinase M2; and GXM—glucuronoxylomannan. This figure was created using elements from Servier Medical Art (www.servier.com) [30,32,35,36,37,38,42,43,44,45,48,50,52].

The studies examined in this review illustrate the wide range of immune responses induced by fungi. MDSCs can play protective roles for the host, as in the case of infections by *C. albicans* [34,39], or they can be a deleterious factor, increasing morbidity and mortality, as in murine infection by *P. brasiliensis* [26]. In 2012, Wu et al. investigated the impact of a polysaccharide from *Lentinus edodes* on MDSCs in a tumor model [33]. The study revealed that the compound was capable of reducing MDSCs in the tumor environment, in addition to suppressing T-cell responses. From then on, different studies began investigating the mechanisms behind this immunosuppressive capacity of MDSCs when stimulated by other species of pathogenic and non-pathogenic fungi. While Table 1 provides a concise summary of the articles underpinning this review, Figure 1 offers a detailed depiction of the mechanisms engaged by MDSCs in response to fungal stimuli or fungi-derived molecules. These mechanisms are further discussed below, organized by context and grouped into thematic sections.

## 2. MDSCs May Play Contradictory Roles When Stimulated by Fungi or Fungal Extracts

As mentioned above, MDSCs are typically generated and accumulate in inflammatory microenvironments, and they have been mostly studied in the context of tumors [10,53]. Despite the scarce research addressing MDSCs in fungal infections specifically, recent studies have begun to explore the role of MDSCs in fungal infection models and their interactions with fungi or fungal molecules in tumor environments. MDSCs serve a dual role, meaning they can be protective or harmful to the host. Some studies have proposed MDSC modulation to enhance future anticancer therapies, particularly when combined with other agents, thus improving therapeutic outcomes. In this context, Li et al. [49] demonstrated in vivo that treatment with a polysaccharide extracted from *Grifola frondosa* can effectively suppress breast tumorigenesis in mice by reducing the accumulation of MDSCs. This effect, primarily in PMN-MDSCs, restored and activated CD8^+^ T-cells through the downregulation of TIGIT (also called T-cell immunoreceptor, a surface protein that suppress T-cell activation [53] expression) and the upregulation of Granzyme B, both known to contribute to the ‘exhaustion’ state of infiltrating T-cells and in enabling CD8^+^ T-cells to eliminate cancer cells, respectively [49].

Previously, Liu et al. [34] also demonstrated the role of MDSCs in murine breast tumor growth, where the use of *Agaricus blazei* Murill polysaccharide (pAbM) inhibited tumor growth in at least two ways: by increasing the production of inducible nitric oxide synthase (iNOS) and arginase-1 from Gr-1^+^ CD11b^+^ monocytic-like MDSCs and by inhibiting the conversion of CD4^+^CD25^−^ T-cells into CD4^+^ Foxp3^+^ CD25^+^ regulatory T-cells (Tregs). In addition, it was demonstrated that pAbM could selectively block the TLR2 signaling in Gr-1⁺ CD11b⁺ MDSCs and enhance their M1-type macrophage characteristics, such as increased IL-12 production, decreased arginase-1 expression, and increased iNOS expression [34].

Similarly, an alternative approach using white button mushrooms (WBMs) as a dietary intervention has been explored [37,43]. WBM, rich in β-glucans, has shown immunomodulatory effects in preclinical studies, reducing MDSCs and enhancing immune responses [47]. A phase I trial in prostate cancer (PCa) patients demonstrated reduced MDSC levels and improved clinical outcomes with WBM consumption [54]. Building on this, a phase II trial was designed to further investigate WBM’s effects using murine models and patient immune cells. The study employed single-cell RNA sequencing and flow cytometry to analyze immune cell changes, revealing that WBM reduces PMN-MDSCs and boosts cytotoxic CD8^+^ T-cell and NK cell activity, slowing tumor progression [47]. Additionally, the profiling of single immune cells from the peripheral blood of patients treated with WBM demonstrated the suppression of STAT3/IRF1 and TGF-β signaling in circulating PMN-MDSCs. Animal studies confirmed WBM’s indirect antitumor effects, mediated by immune system modulation rather than direct cytotoxicity. WBM also enhanced the efficacy of immunotherapy, such as PD-1 inhibitors, by increasing tumor regression and survival outcomes, indicating WBM as a complementary treatment alongside immune checkpoint inhibitors [47].

Concerning fungal extracts, the *Ganoderma microsporum* immunomodulatory peptide (GMI), a fungal immunomodulatory peptide isolated from *Ganoderma microsporum* (NCBI protein ID AGU04723.1), suppresses the expansion of MDSCs, favoring the proliferation of cytotoxic T-cells during infection in mice [41]. Normally, the formation of methicillin-resistant *Staphylococcus aureus* (MRSA) biofilms promotes the expansion of MDSCs, thus inhibiting the proliferation of cytotoxic T-cells, by favoring bacterial infections. Interestingly, treatment with GMI increased the production of IL-6 and TNF-α, concomitantly with a reduction in ROS and inhibition of MDSCs in whole blood and in the bone region in mouse models of periprosthetic joint infection caused by *S. aureus* [30].

In response to fungal infection, MDSCs represent an essential mechanism of immunological modulation, as initially demonstrated by Rieber et al. [14], where infections by *A. fumigatus* and *C. albicans* promoted the emergence of a distinct myeloid cell population, characterized by the expression of CD33, CD11b, CD16, and CXCR4. This population differed from conventional monocytes and exhibited a potent capacity to suppress the proliferation of CD4⁺ and CD8⁺ T-cells, thereby confirming its identity as MDSCs. A relevant aspect identified in the study is the ability of these fungi-induced MDSCs to suppress the response of NK cells without compromising their cellular viability [14]. This suppressive effect differs from the function of MDSCs induced by growth factors, which traditionally modulate immune responses distinctly. Notably, the MDSCs induced by fungal infections also demonstrated an impact on attenuating Th2 responses, suggesting a potential role in the disease regulation associated with fungal hypersensitivity, such as fungal asthma [54] MDSC characterization as predominantly neutrophilic, without a significant presence of the monocytic subtype, represented an important distinction concerning other inflammatory and infectious conditions. The functionality of these cells, evidenced by their suppression of T-cell proliferation, contrasts with the activity of conventional neutrophils, which do not exhibit this property. These data reinforce the essential role of MDSCs in balancing the protective immune response and immunosuppression, thereby preventing excessive tissue damage [35,36]. Thus, the induction of MDSCs by pathogenic fungi stood out as a critical mechanism for regulating the immune response, influencing the prognosis of infections.

Studies investigating MDSCs in candidiasis have yielded significant findings regarding the induction of neutrophilic cells by *Candida* species through PRRs. Notably, Singh et al. conducted an in vitro experimental study using peripheral blood myeloid cell samples co-cultured with fungi from various species within this taxonomy to confirm the PRR-mediated induction of PMN-MDSCs. The results showed that, during the pathogenesis of infections by *C. albicans*, Dectin-1, in association with CARD9, promoted a reduction in CD4⁺ and CD8⁺ T-lymphocytes and greater secretion of GM-CSF, contributing to the induction of PMN-MDSCs when compared to other species, namely, non-*albicans Candida* species. Dectin-2 did not yield similar results. Furthermore, the experiment revealed that *C. glabrata* and *C. krusei* exhibited greater immunosuppression of T-cells and NK cells compared to *C. albicans*. In contrast, the opposite effect was observed in *Candida parapsilosis* and *Candida dubliniensis* [36].

In vivo studies using immunocompromised mice discussed the relationship between G-MDSCs and the inhibition of the protective neutrophil response against oropharyngeal candidiasis (OPC). This analysis alludes to the fact that neutrophils form “swarms” via the activation of the LTB4R1/BLT1 complex through IL-1 and G-CSF signaling from connective tissue in a chemotactic process toward the site of infection. Accordingly, when associated with *Candida albicans* hyphae, the G-MDSCs release arginase-1, which inhibits the activation of the LTB4R1/BLT1 complex. Consequently, the phagocytosis of pathogens by neutrophils is compromised, which reveals the contribution of G-MDSCs to the progression of OPC [44].

There are also experimental studies that have demonstrated the influence of G-MDSCs in *A. fumigatus* infection. In this regard, Mueller-Leisse et al. showed that this granulocytic type of suppressive cell compromises the clearance of *A. fumigatus* by NK cells, caused by a decrease in IFN-γ production by these leukocytes. Thus, compared to the normal clearance and cytotoxicity of *A. fumigatus* in control groups, NK cell inhibition was significantly greater in co-cultures with G-MDSCs due to the decreased expression of surface receptors NKp30 and proteins CD69 and CD137, which target leukemic cells of the K562 type [35].

## 3. MDSCs Worsen the Disease in *Paracoccidioides brasiliensis* Infection

Paracoccidioidomycosis (PCM) is a chronic systemic mycosis caused by thermally dimorphic fungi, including *P. brasiliensis*, *Paracoccidioides lutzii*, and other species like *Paracoccidioides americana*, *Paracoccidioides restrepiensis*, and *Paracoccidioides venezuelensis* [55]. Endemic to Latin America, particularly Brazil, Colombia, and Venezuela, PCM has the highest mortality rate among systemic mycoses (1.45 per million inhabitants) and is considered an occupational disease due to its prevalence among agricultural workers [56]. Infection typically occurs via the inhalation of mycelial fragments or conidia, primarily affecting the lungs and leading to complications due to primary infection or the reactivation of latent foci [57,58]. Immunologically, resistance to PCM is linked to Th1 cytokine responses, including IFN-γ, which activates macrophages, while a predominant Th2 cytokine profile correlates with systemic and progressive disease [59,60]. Th17 responses, implicated in granuloma formation, also play a role in PCM, although their exact contribution remains unclear [60]. Variations in T-cell responses influence clinical outcomes, with asymptomatic individuals showing a dominant Th1 response, while severe forms of PCM are associated with Th2 or Th2/Th9 profiles and higher antibody production [61]. Furthermore, immunosuppressive mechanisms, including tolerogenic plasmacytoid dendritic cells (pDCs), the enzyme IDO-1, and Tregs, are critical in PCM pathogenesis [8,62]. Studies have shown that the depletion of Tregs improves immunity and prevents fatal outcomes in murine models [63], while an increased presence of FoxP3^+^ Tregs in PCM patients correlates with disease severity [64,65]. Additionally, the immunosuppressive role of pDCs, through IDO-1 and Tregs, exacerbates pulmonary PCM severity, as seen in other fungal infections like candidiasis and aspergillosis [8,66].

In 2023, Preite et al. [20] showed that monocytic-like and polymorphonuclear-like MDSCs expressing PD-L1, IL-10, IDO-1, and NO extensively infiltrated the lungs during murine *P. brasiliensis* infection. A partial reduction in MDSC frequency with anti-Gr1 treatment led to a pronounced Th1/Th17 lymphocyte response, contributing to disease regression, a lower fungal burden in target organs, reduced pulmonary pathology, and decreased mortality compared to IgG2b-treated control mice [20]. The immunosuppressive effects of MDSCs on CD4^+^ and CD8^+^ T-cells, as well as Th1/Th17 cells, were confirmed through in vitro coculture experiments. In contrast, the adoptive transfer of MDSCs into *P. brasiliensis*-infected recipient mice resulted in aggravated disease severity [20]. Collectively, these findings indicate that an increased influx of MDSCs into the lungs is associated with more severe disease and suppression of Th1 and Th17 protective responses. However, anti-Gr1 treatment restored protective immunity, leading to milder disease and improved tissue pathology control. Thus, MDSCs have emerged as potential targets for adjuvant therapy in PCM [20].

Subsequently, Preite et al. [51] investigated MDSC depletion as a therapeutic strategy for PCM using the chemotherapy drug 5-Fluorouracil (5-FU), demonstrating a reduction in pulmonary MDSCs and fungal burden. The targeted depletion of MDSCs led to a decrease in all pulmonary CD4^+^ T-cell populations, which correlated with improved tissue pathology and increased survival [51]. This reduction was associated with higher frequencies of Th1/Th17 cells and elevated levels of Th1/Th2/Th17 cytokines in the lungs and liver of treated mice, indicating an early and effective protective immune response. Additionally, the protective immunity induced by 5-FU treatment was reversed by MDSC adoptive transfer [51]. These findings suggest that 5-FU-mediated MDSC depletion enhances immunity in *P. brasiliensis*-infected mice and represents a promising immunotherapeutic approach for PCM. The same approaches were reported by Green et al. [67], who, in the context of an LP-BM5 retroviral infection, treated mice with 5-FU. The treatment resulted in a decreased frequency of M-MDSCs in both peripheral blood and the spleen, accompanied by a significant elevation in CD4^+^ and CD8^+^ T-cells, restoring T-cell function and improving the protective immune response.

Another study revealed that IDO-1 expression by MDSCs plays a significant role in regulating T-cell proliferation, with partial dependence on Dectin-1, TLR2, and TLR4 signaling in pulmonary PCM [30]. Further addressing the role of PRRs in the suppressive activity of MDSCs, our group has also demonstrated that Dectin-1, TLR2, and TLR4 enhance the suppressive function of MDSCs by promoting the expression of immunosuppressive molecules, including PD-L1, IL-10, and nitrotyrosine—a compound resulting from the reaction between NO and superoxide, producing peroxynitrite, which can be detected with an anti-nitrotyrosine antibody [68]. The study provided the first evidence of a complex network of PRR signaling involved in inducing multiple suppressive molecules in MDSCs, highlighting its role in immunosuppressive mechanisms that regulate immune responses and influence the severity of pulmonary PCM in mice.

## 4. MDSC Recruitment by *Cryptococcus neoformans* Worsens Host Defense

Cryptococcosis is a severe systemic mycosis caused by the encapsulated yeasts *C. neoformans* and *C. gattii*, typically acquired from environmental sources. In Latin America, similar to other parts of the world, *C. neoformans* is responsible for over 90% of cryptococcosis cases, primarily affecting individuals with HIV, while *C. gattii* tends to infect otherwise healthy individuals [69]. The immune response to *C. neoformans* infection is characterized by a complex interplay between innate and adaptive immunity, with a crucial role played by alveolar macrophages, DCs, and T-helper lymphocytes. The polysaccharide capsule of *C. neoformans*, primarily composed of glucuronoxylomannan (GXM), inhibits phagocytosis and modulates host immune responses by inducing anti-inflammatory cytokine production, such as IL-10, while suppressing pro-inflammatory cytokines like TNF-α and IFN-γ. Effective fungal clearance requires a Th1-skewed response, where IFN-γ and TNF-α activate macrophages, promoting fungal killing through NO and ROS. Conversely, a Th2-dominant response is associated with poorer outcomes, as IL-4 and IL-13 facilitate alternative macrophage activation, leading to fungal persistence [70]. In immunocompromised individuals, particularly those with HIV/AIDS (Acquired Immunodeficiency Syndrome), impaired CD4^+^ T-cell responses hinder the development of protective immunity, increasing susceptibility to disseminated cryptococcosis and meningoencephalitis [71].

In this context, a study conducted by Li et al. [32] investigated the immunomodulatory effects associated with the manipulation of the enzyme arginase-1, using mice experimentally infected with *C. neoformans*. The researchers applied inhibitors such as p38 and SB202190, combined with T-cell-based immunotherapy. In vitro activation restored T-cell functionality before reintroduction into the animals. Initially, infection with *C. neoformans* promoted the recruitment of MDSCs, particularly the granulocytic subpopulation. This recruitment resulted in the inhibition of antifungal T-cell-mediated immunity and an increased fungal burden, as evidenced by the use of an anti-Ly6G antibody, which induced PMN-MDSC depletion and, consequently, a significant reduction in fungal burden and an increased survival rate in mice. Additionally, the study revealed that GXM, a component of the *C. neoformans* capsule, induced the recruitment of PMN-MDSCs to the lungs of infected mice. This recruitment led to a dose-dependent suppression of CD4^+^ and CD8^+^ T-cell proliferation. The underlying mechanism of GXM’s effect involved blocking the interaction between the CLEC2D and *C. neoformans*. This interaction was crucial for inhibiting the cytotoxic function of T-cells and NK cells, promoting the immunosuppressive environment induced by infection. As stated above, similar findings were reported in the study by Preite et al. [20], which addressed *P. brasiliensis* infection. These studies underscore the pivotal role of MDSCs in modulating immune responses during fungal and viral infections and suggest that targeting their function or recruitment may represent a promising strategy for treating infectious diseases.

Recently, a study conducted in a mouse model demonstrated that infection with either the B3501 or CAP67 strain of *C. neoformans* leads to the accumulation of granulocytic MDSCs in the bronchoalveolar spaces. Interestingly, MDSCs elicited by the B3501 strain were capable of suppressing T-cell proliferation, whereas those induced by the CAP67 strain did not display such suppressive properties. Moreover, PD-L1 expression was identified on these cells, pointing to a potential mechanism of immune regulation during cryptococcal infection. These findings suggest that the polysaccharide components of *C. neoformans* may facilitate immune evasion by undermining the host’s immune response [52].

## 5. Fungi-Induced MDSCs Can Play a Role in the Fight Against Cancer Tumors

Colorectal cancer (CRC) is the third most common cancer and the second most common cause of cancer mortality worldwide. In 2020, more than 1.9 million new cases occurred, and almost 0.9 million patients died worldwide [72]. It is well known that intestinal dysbiosis is associated with the onset and progression of colitis and CRC [73,74]. The development of inflammatory bowel disease also involves dysbiosis of the fungal microbiome and bacteria [75]. Considering the importance of the Dectin-1 receptor for the recognition of fungi by the innate immune system and the role of this receptor in the activity of MDSCs in fungal infections [14], it has been shown that inhibiting Dectin-1 halts colorectal tumor development by reducing prostaglandin E2 production in MDSCs and increasing the expression of IL-22 binding protein [76].

In mice lacking Dectin-1, the abundance of opportunistic pathogenic fungi such as *C. tropicalis* increases considerably during DSS-induced colitis, aggravating the condition [76]. Zhang et al. [42] isolated bone marrow-derived MDSCs and stimulated them with *C. tropicalis*, which increased the immunosuppressive capacity of MDSCs, with an increase in the production of iNOS, COX2, NOX2, NO, and ROS through the C-type lectin receptors Dectin-3 and Syk. On the one hand, NO produced by MDSCs increased aerobic glycolysis. On the other hand, *C. tropicalis* promoted the binding of p-Syk to PKM2, which resulted in the phosphorylation of PKM2 Tyr105 and the nuclear translocation of PKM2 in MDSCs. Nuclear PKM2 interacted with HIF-1α and upregulated the expression of HIF-1α target genes encoding glycolytic enzymes, including GLUT1, HK2, PKM2, LDHA, and PDK1, which are required for the aerobic glycolysis of *C. tropicalis*-induced MDSCs. Blocking PKM2 nuclear translocation attenuated fungi-mediated colorectal tumorigenesis. Overall, the study showed that high expression of PKM2, PKM2 (p-Y105), and iNOS in CRC-infiltrated MDSCs correlates with the development of human CRC.

Further analysis of clinical samples from CRC indicated that high levels of IL-1β expression were closely linked to the immunosuppressive functions of tumor-infiltrating MDSCs due to a significant correlation between *IL1B* mRNA expression and the genes Nos2, Ptgs2, and Cybb, which are closely related to the immunosuppressive activity of MDSCs. In vitro, IL-1β was the most abundantly secreted cytokine by MDSCs when stimulated by *C. tropicalis*, contributing to cancer progression. Interestingly, adding IL-1β in vitro further boosted the immunosuppressive properties of MDSCs stimulated by *C. tropicalis* [48]. Of note, the NLRP3-IL-1β signaling pathway mediated this enhancement of MDSC immunosuppression, and the blockade of IL-1β reversed the immunosuppressive stimulation promoted by the fungus, decreasing the expression of iNOS, COX2, and NOX2. Finally, inhibiting the secretion of IL-1β from MDSCs increased antitumor immunity, restoring CD8^+^ T-cell infiltration in the spleen and tumors and reducing the effects of *C. tropicalis*-associated colon cancer [48]. Notably, MDSCs pretreated with *C. tropicalis* stimulated lower expression of Granzyme B and IFN-γ and higher PD-1 expression in tumor-bearing mice. This effect disappeared after the IL-1β blockage [48].

As an attempt to explore the role of the intratumor mycobiome in cancer progression, a study used fungi-enriched DNA extraction and deep shotgun metagenomic sequencing to identify enriched tumor-resident *Aspergillus sydowii* in patients with lung adenocarcinoma (LUAD). Interestingly, the results showed that *A. sydowii* promoted tumor progression via IL-1β-mediated expansion and the activation of MDSCs, resulting in suppressed activity of cytotoxic T-cells and an accumulation of PD-1^+^ CD8^+^ T-cells. This elegant study supported the inclusion of fungi in the updated cancer microbiome “hallmarks” and suggested their potential as prognostic markers and therapeutic targets in lung cancer [45].

## 6. MDSCs Act as Drivers of the “Trained Tolerogenic Immunity” Induced by Low-Virulence Fungi Infection

Combined effects have been reported between *Candida* spp. and multiple bacterial taxa, including representatives from both Gram-positive and Gram-negative classes. Yamabayashi et al. [77] reported that mixed inoculations of *C. albicans* with *Proteus vulgaris* or *Pseudomonas aeruginosa* caused increased mortality in mice. Similar synergism has been reported for *Mycobacterium tuberculosis* as well as enteric pathogens, including *S. aureus*, *Serratia marcescens, Streptococcus faecalis*, *Escherichia coli,* and *E. coli*/*Bacteroides fragilis* [78,79,80,81,82,83]. The role of MDSCs in these cases is also being investigated. Synergistic interactions between fungi and bacteria, such as *C. albicans* and *S. aureus*, lead to high mortality in murine polymicrobial infections, while *C. dubliniensis* protects against these lethal infections. This protection is driven by a novel form of trained innate immunity (TII), called “trained tolerogenic immunity” (TTI), mediated by MDSCs. While MDSCs are typically harmful in sepsis, their role can be protective, as seen in certain sepsis models. A 2019 review by Esher et al. [83] explored the role of MDSCs in sepsis and infection, emphasizing their contribution to trained immune protection against fungal–bacterial sepsis. Polymicrobial infections, particularly those involving fungi, are increasingly prevalent and challenging to treat. A novel TTI induced by *C. dubliniensis* that protects against fungal–bacterial intra-abdominal infections (IAIs) has been proposed, in which MDSCs play a key role in this protection. It was proposed that MDSC-mediated protection in polymicrobial sepsis falls within the spectrum of trained innate memory, where the suppression of pathological inflammation represents TTI. Future research should focus on elucidating how MDSCs develop in response to *C. dubliniensis* and their role in protecting polymicrobial intra-abdominal infection (IAI) models.

Rieber et al. [14] also showed that fungi-induced MDSCs, which were protective against candidiasis, exhibited enhanced antifungal activity. However, they concluded that such antifungal activity played a relatively minor role in overall protection. One potential explanation for the protection against IAIs is that *C. dubliniensis*-trained MDSCs suppress the septic response and differentiate into other innate immune cells, ultimately clearing *C. albicans*/*S. aureus*. Several studies have indicated that MDSCs retain the ability to differentiate into mature innate cells [84,85]. Another possibility is that primary protection results from the suppression of the MDSC-mediated septic response, while antimicrobial activity is driven by a classic innate immune response, namely, polymorphonuclear neutrophils and macrophages. In this context, if the lethal septic inflammatory response is suppressed, classic innate cells would have sufficient time to function effectively and reduce the infection source [83].

Lilly et al. [37,40] investigated the breadth of trained innate immunity (TII) induced by low-virulence *Candida* species—particularly *C. dubliniensis*—demonstrating protective effects in both lethal polymicrobial sepsis and vaccination-induced intra-abdominal infection (IAI) models, with a central role for bone marrow infiltration and recruitment of MDSCs in mediating this response. The literature defines this low-virulence species as *C. dubliniensis* [86]. First, several microbial requirements of TII were identified in the protective response against polymicrobial IAI/sepsis. Compared to the protection conferred by controls that received only the lethal *C. albicans*/*S. aureus* challenge, each fungal species tested in combination with *S. aureus* conferred significant protection against the lethal *C. albicans*/*S. aureus* challenge. However, the most significant finding was the infiltration of several fungal species into the bone marrow following a primary intraperitoneal (IP) challenge. This finding is particularly important because of its direct connection to the recruitment of MDSCs and TII, rather than being an isolated observation. Fungal infiltration into the bone marrow had not been extensively studied at that time [37].

In a following study, IP-vaccinated mice were protected against lethal sepsis following *C. albicans*/*S. aureus* IAI infection or *C. albicans* bloodstream infection (BSI). Protection against IAI was mediated by long-lived Gr-1^+^ leukocytes such as MDSCs and not by prototypic-trained macrophages. Gr-1 is a surface receptor and consists of Ly6G and Ly6C antigens. Therefore, Gr-1 antibody depletion affects Ly6G^+^ granulocytic cells (neutrophils and PMN-MDSCs) and Ly6C^+^ monocytic cells (monocytes and M-MDSCs). Vaccinated animals were treated with anti-Ly6G antibodies similar to Gr-1^+^ cell depletion before and during the lethal challenge period. In each model, the depletion of Ly6G^+^ cells abrogated vaccination-mediated protection, as evidenced by increased mortality compared to control animals. In addition, MDSC populations in the blood and spleens of vaccinated mice confirmed that both Ly6G and Gr-1 antibody treatment specifically depleted G-MDSCs [40].

In addition, it is known that MDSCs are involved during episodes of sepsis. The study by Harriett et al. [43] demonstrated that Gr-1^+^ MDSCs play an essential role in protecting against fungal and bacterial sepsis through TII. The IP inoculation of low-virulence *C. dubliniensis* in mice protected against infections caused by *C. albicans* and *S. aureus* and reduced pro-inflammatory cytokine levels. To evaluate the extent of this protection, mice were immunized, and after 14 days, they were challenged with lethal infectious agents. The prominence of this protection was highlighted due to the performance of Gr-1^+^ MDSCs. In immunized mice with a depletion of Gr-1^+^ cells via anti-Gr-1 antibody administration, the survival rate was comparable to that of the control groups.

Similar results were reported in mice immunized by modified β-glucan and submitted to a depletion of Gr-1^+^ cells, which exhibited a survival rate of 10% after 3 days of infection. The anti-Gr-1-mediated depletion, which affects both Ly6G and Ly6C, predominantly associated with G-MDSCs and M-MDSCs, respectively, highlights the importance of both cell subtypes in immune response regulation against fungal sepsis. β-glucan alone has been shown to have immunomodulatory effects by stimulating innate and adaptive immune responses and cells, including MDSCs. Functional assays indicated that MDSCs from β-glucan-treated neonates presented impaired immunosuppressive function compared to controls. Specifically, both ROS levels and arginase-1 expression were reduced in β-glucan-derived PMN-MDSCs. Furthermore, the administration of β-glucan significantly decreased the frequency and ROS levels of PMN-MDSCs in vitro. These findings reinforced that β-glucan plays a role in promoting the maturation of myeloid cells in early life, which may help protect against immune disorders in later stages [46].

## 7. Conclusion Remarks and Therapeutic Perspectives

MDSCs demonstrate a complex and, occasionally, contradictory role in fungal infections. Some pathogens, such as *P. brasiliensis* and *C. neoformans*, can induce the recruitment and expansion of MDSCs, exacerbating disease severity through immunosuppressive mechanisms. In *P. brasiliensis* infection, MDSCs contribute to worsening the disease by impairing T-cell responses, while in *C. neoformans* infection, PMN-MDSCs hinder antifungal immunity, allowing fungal persistence and exacerbating conditions like meningoencephalitis [14,32]. Beyond infectious diseases, fungi-induced MDSCs have also been implicated in tumor progression [42,45]. In certain cancers, such as colorectal cancer, MDSCs stimulated by fungal interactions, like those involving *C. tropicalis*, can modulate the immune environment, contributing to tumor progression. These findings highlight that the dual roles of MDSCs—acting as both detrimental and protective cells—are context-dependent and influenced by the pathogen or tumor microenvironment [42,45].

Furthermore, fungi can induce a “trained tolerogenic immunity,” where low-virulence fungal infections shape MDSCs into immunosuppressive regulators, enhancing tolerance and contributing to long-term immune modulation. This phenomenon suggests that fungi can promote a unique immune regulation, potentially leveraging MDSCs to prevent harmful immune responses while tolerating non-pathogenic organisms. These complex interactions between fungi, MDSCs, and the immune system underscore the need for a deeper investigation to better understand the nuanced roles of MDSCs in both fungal infections and cancer, providing avenues for therapeutic targeting and immune modulation [45,49,53,67,75].

**Figure 2:**
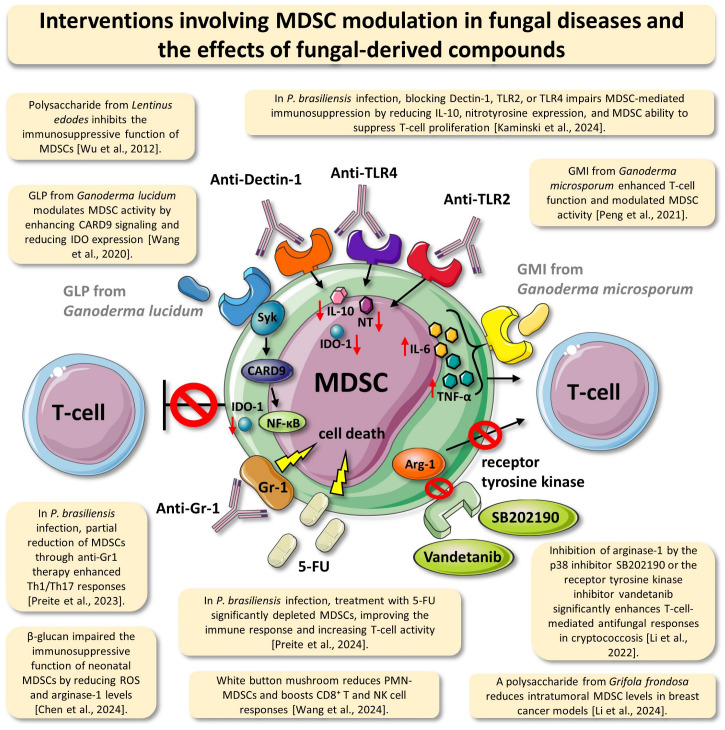
Interventions involving MDSC modulation in fungal diseases and the effects of fungal compounds. This figure illustrates the mechanisms by which various therapeutic interventions—including bioactive compounds derived from fungi, synthetic drugs, and immunotherapeutic strategies—modulate the proliferation and function of MDSCs in the context of fungal infections. Natural agents such as polysaccharides from *Lentinus edodes*, *Ganoderma lucidum* (GLP), *Ganoderma microsporum* (GMI), β-glucan, white button mushroom (*Agaricus bisporus*), and *Grifola frondosa* have been shown to reduce MDSC immunosuppressive activity by modulating intracellular signaling pathways (e.g., CARD9, NF-κB, and TLRs) or by inhibiting the expression of enzymes such as indoleamine 2,3-dioxygenase 1 (IDO-1) and arginase-1. Additionally, pharmacological approaches using 5-fluorouracil (5-FU), anti-Gr-1 antibody, tyrosine kinase inhibitors (e.g., Vandetanib), or p38 inhibitors (e.g., SB202190) promote MDSC depletion, cell death, or the restoration of T-cell function. These strategies, investigated in murine models of infection with *Paracoccidioides brasiliensis*, *Cryptococcus neoformans*, and others, highlight the potential of targeting MDSCs as a therapeutic approach in fungi-induced immunosuppression. Selected findings from the literature supporting these interactions are presented in the annotated boxes surrounding the central MDSC. Abbreviations: MDSC—myeloid-derived suppressor cell; GLP—*Ganoderma lucidum* polysaccharide; GMI—*Ganoderma microsporum* immunomodulatory peptide; IL—interleukin; IDO-1—indoleamine 2,3-dioxygenase 1; Arg-1—arginase-1; TLR—Toll-like receptor; CARD9—caspase recruitment domain-containing protein 9. This figure was created using elements from Servier Medical Art (https://smart.servier.com) [20,32,33,39,40,46,47,49,50,51].

Of note, the modulation of MDSCs presents a promising avenue for enhancing immune responses in oncological and infectious diseases, particularly when induced by fungal components. Various studies have demonstrated that targeting MDSCs can potentiate anticancer therapies, as evidenced by the immunomodulatory effects of fungal polysaccharides, such as those derived from *G. frondosa*, which enhance cytotoxic CD8^+^ T-cell function and improve tumor control [49]. Additionally, interventions that deplete MDSCs, such as 5-FU treatment, have been shown to restore protective immunity and reduce fungal burden in *P. brasiliensis* infection, reinforcing the role of MDSCs as key immunoregulatory players [51]. Interventions involving MDSC modulation in fungal diseases, as well as the modulation of MDSCs by fungal compounds, are illustrated in Figure 2.

Moreover, recent insights into fungi-induced immunosuppression have revealed novel therapeutic targets, such as the arginase-1 pathway in *C. neoformans* infections, where the inhibition of MDSC recruitment enhances T-cell responses [32]. Similarly, the identification of tumor-resident *A. sydowii* in lung adenocarcinoma has unveiled a direct link between fungal elements, MDSC expansion, and immune evasion through IL-1β signaling [45]. These findings collectively support the therapeutic potential of modulating MDSCs in malignancies and fungal infections.

Future studies should explore the precise molecular mechanisms by which fungal components drive MDSC differentiation and function, focusing on uncovering new immunotherapeutic targets. Advances in single-cell transcriptomics and spatial immune profiling may provide deeper insights into the heterogeneity of MDSCs in fungal infections and cancer, facilitating the development of more selective therapeutic strategies. Furthermore, investigating the interplay between the microbiome, fungal colonization, and MDSC-mediated immune modulation could open new avenues for microbiota-based therapies. Recent calls for novel strategies to combat pathogenic fungi, including *Candida auris*, underscore the critical need to thoroughly understand all potential antifungal therapeutic approaches that are effective against fungal cells and safe for humans. These warnings highlight the urgency of developing and evaluating treatment options that can address the growing threat posed by such resistant fungi [87]. Ultimately, integrating fungal immunomodulators with emerging checkpoint inhibitors or adoptive cell therapies may enhance treatment efficacy, offering novel immunotherapeutic approaches for infectious diseases and malignancies.

## Figures and Tables

**Table 1:** Studies addressing MDSCs and fungi. The articles are organized chronologically.

Type of Study	Main Findings	Reference
In vivo and in vitro	MPSSS from *Lentinus edodes* reduced MDSCs in tumor-bearing mice, enhanced CD4⁺ T-cell activation, and altered MDSC signaling, promoting a stronger immune response.	[33]
In vivo and in vitro	*A. fumigatus* and *C. albicans* induced MDSC recruitment, which suppressed T-lymphocytes and NK cells. MDSC recruitment was mediated by Dectin-1 and IL-1β. In addition, MDSC transfer improved survival in *C. albicans* infection but not in *A. fumigatus*.	[14]
In vivo	Polysaccharides from *Agaricus blazei* Murill promoted the differentiation of MDSCs from an M2 to M1 phenotype, helping to inhibit tumor immune evasion via the TLR2 pathway.	[34]
In vitro	Granulocytic MDSCs and polymorphonuclear neutrophils inhibited NK cell activity toward *Aspergillus fumigatus*.	[35]
In vitro	Compared to *C. albicans*, *C. krusei* and *C. glabrata* more potently induced MDSC-mediated immunosuppression via Dectin-1. MDSC recruitment was driven by GM-CSF and IL-1β, resulting in NK cell inhibition.	[36]
In vivo	Co-infection with *C. albicans* and *Staphylococcus aureus* led to high mortality due to excessive inflammation. In contrast, previous infection with low-virulence *C. dubliniensis* protected against lethality by expanding and activating MDSCs, suggesting a potential vaccine strategy.	[37]
In vivo	CARD9 deficiency promoted immunosuppression, tumor progression, and colorectal cancer susceptibility by increasing MDSCs, inducing dysbiosis, and impairing immune regulation.	[38]
In vivo	*Ganoderma lucidum* polysaccharides (GLPs) boosted antitumor immune responses by modulating the differentiation and suppression of MDSCs through the CARD9–NF-κB–IDO signaling pathway.	[39]
In vivo	Innate immune training induced by vaccination with low-virulence *Candida* species conferred protection against diverse forms of fungal sepsis through Ly6G⁺ Gr-1⁺ myeloid cells (which the authors referred to as putative MDSCs).	[40]
In vivo	Treatment with GMI, an immunomodulatory peptide from *Ganoderma microsporum*, enhanced T-cell function and modulated MDSC activity, improving the immune response against *G. microsporum* infection.	[41]
In vivo and in vitro	*Candida tropicalis* stimulation enhanced iNOS, COX2, and NOX2 expression in MDSCs, with iNOS-derived nitric oxide activating their glycolytic metabolism; the inhibition of PKM2 reduced these markers, underscoring the role of *C. tropicalis* and PKM2 in MDSC metabolic activation.	[42]
In vivo	The inhibition of arginase-1 production by MDSCs enhanced T-cell-based immunotherapy against *C. neoformans* infection.	[32]
In vivo	Immunization with *C. dubliniensis* and β-glucan protected against sepsis induced by *C. albicans* and *Escherichia coli*. Such protection was mediated by Gr-1^+^ cells (MDSCs). In addition, Gr-1^+^ cell depletion increased mortality, with varying β-glucan efficacy depending on the pathogen.	[43]
In vivo	PMN-MDSCs associated with neutrophil swarms played a key role in resolving oropharyngeal candidiasis by preventing deep invasion of *C. albicans* into the oral mucosa.	[44]
In vivo	Monocytic and polymorphonuclear MDSCs infiltrated the lungs during *Paracoccidioides brasiliensis* infection. Partial depletion of MDSCs through anti-Gr1 therapy enhanced Th1/Th17 responses, resulting in disease regression, reduced fungal burden, less lung pathology, and lower mortality compared to the control group.	[20]
In vivo	In *P. brasiliensis* infection, IDO-1 expression by MDSCs regulated T-cell proliferation. IDO-1 production by MDSCs partially depended on Dectin-1, TLR2, and TLR4 signaling during murine paracoccidioidomycosis.	[30]
In vivo	In lung adenocarcinoma, *Aspergillus sydowii* promoted MDSC accumulation through IL-1β induction, contributing to an immunosuppressive microenvironment and tumor progression.	[45]
In vivo and in vitro	β-glucan impaired the immunosuppressive function of MDSCs in neonates, reducing ROS and arginase-1 levels. It also decreased the frequency and ROS levels in PMN-MDSCs in vitro.	[46]
In vivo	White button mushroom (WBM) inhibited tumor growth in prostate cancer murine models by reducing PMN-MDSCs and inhibiting the STAT3/IRF1 and TGFβ pathways. In patients, treatment decreased PMN-MDSCs and increased CD8⁺ T-cells and NK cells. WBM enhanced anti-PD-1 antibody efficacy.	[47]
In vivo and in vitro	Stimulation of MDSCs with *C. tropicalis* increased IL-1β expression, enhancing their activity and promoting cancer progression. In vivo, *C. tropicalis* infection increased tumor burden and elevated the MDSC levels and activity.	[48]
In vivo	A polysaccharide from *Grifola frondosa* reduced the frequency of MDSCs in the tumor microenvironment and inhibited tumor growth in breast cancer models. The treatment also enhanced T-cell responses, potentiating immune activity against tumor cells.	[49]
In vivo and in vitro	In *P. brasiliensis* infection, IDO-1 expression by MDSCs regulated T-cell proliferation, with IDO-1 production being partially dependent on Dectin-1, TLR2, and TLR4 signaling.	[50]
In vivo	In *P. brasiliensis* infection, treatment with 5-FU depleted MDSCs, improving the immune response by increasing T-cell activity and enhancing pro-inflammatory cytokine production, reducing disease severity.	[51]
In vivo	In *C. neoformans* infection, MDSCs induced by the B3501 strain exhibited T-cell suppressive activity, whereas those associated with the CAP67 strain lacked this function.	[52]

## Data Availability

No new data were created or analyzed in this study. Data sharing is not applicable to this article.
